# Phenylphosphonate surface functionalisation of MgMn_2_O_4_ with 3D open-channel nanostructures for composite slurry-coated cathodes of rechargeable magnesium batteries operated at room temperature

**DOI:** 10.1039/d1ra02598h

**Published:** 2021-05-26

**Authors:** Koichi Kajihara, Daisuke Takahashi, Hiroaki Kobayashi, Toshihiko Mandai, Hiroaki Imai, Kiyoshi Kanamura

**Affiliations:** Department of Applied Chemistry for Environment, Graduate School of Urban Environmental Sciences, Tokyo Metropolitan University 1-1 Minami-Osawa Hachioji Tokyo 192-0397 Japan kkaji@tmu.ac.jp; Institute of Multidisciplinary Research for Advanced Materials, Tohoku University 2-1-1 Katahira, Aoba-ku Sendai Miyagi 980-8577 Japan; Center for Green Research on Energy and Environmental Materials, National Institute for Materials Science (NIMS) 1-1 Namiki Tsukuba Ibaraki 305-0044 Japan; Department of Applied Chemistry, Faculty of Science and Technology, Keio University 3-14-1 Hiyoshi, Kohoku-ku Yokohama Kanagawa 223-8522 Japan

## Abstract

Spinel-type MgMn_2_O_4_, prepared by a propylene-oxide-driven sol–gel method, has a high surface area and structured bimodal macro- and mesopores, and exhibits good electrochemical properties as a cathode active material for rechargeable magnesium batteries. However, because of its hydrophilicity and significant water adsorption properties, macroscopic aggregates are formed in composite slurry-coated cathodes when 1-methyl-2-pyrrolidone (NMP) is used as a non-aqueous solvent. Functionalising the surface with phenylphosphonate groups was found to be an easy and effective technique to render the structured MgMn_2_O_4_ hydrophobic and suppress aggregate formation in NMP-based slurries. This surface functionalisation also reduced side reactions during charging, while maintaining the discharge capacity, and significantly improved the coulombic efficiency. Uniform slurry-coated cathodes with active material fractions as high as 93 wt% can be produced on Al foils by this technique employing carbon nanotubes as an electrically conductive support. A coin-type full cell consisting of this slurry-coated cathode and a magnesium alloy anode delivered an initial discharge capacity of ∼100 mA h g^−1^ at 25 °C.

## Abstract 2

Phenylphosphonate functionalisation is an easy, highly effective strategy to fabricate slurry-coated nanostructured MgMn_2_O_4_ cathodes for rechargeable magnesium batteries at active material fractions up to 93 wt% for rechargeable magnesium batteries cycled at 25 °C.

## Graphical abstract

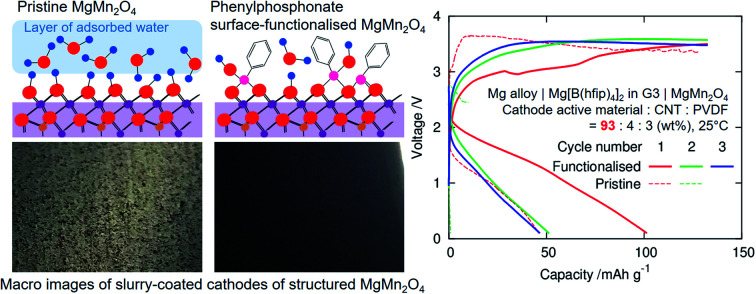

## Introduction

Rechargeable magnesium batteries (RMBs) have attracted attention because Mg has high natural abundance and is safer to handle than Li in metal anodes, and the divalent mobile Mg^2+^ ions can increase the energy density of active materials.^1–4^ Promising cathode materials for RMBs include transition metal spinel oxides, such as AB_2_O_4_ (A = Mg, Zn, Mn; B = Mn, Fe, Co, Ni)^5–23^ and ZnMnO_3_.^24^ These compounds discharge at an operating voltage of ∼2–3 V *vs.* Mg/Mg^2+^ along with Mg^2+^ ion insertion and simultaneous transformation into Mg-rich rock-salt-like phases.^8,9,22^ Although aqueous electrolytes have commonly been used in electrochemical reactions of Mg^2+^ ions with spinel-type cathodes,^5,6,10–13^ non-aqueous electrolytes are required to achieve high-voltage operation with metallic Mg anodes.^2,8,9^ Several studies using non-aqueous electrolytes have been conducted at elevated temperatures (≥100 °C)^8,9,15–22,24^ to facilitate the transport of Mg^2+^ ions by overcoming the strong coulombic interactions between Mg^2+^ ions and the host oxide lattice.

A general approach to enhance the insertion and extraction of Mg^2+^ ions is to reduce the particle size of host lattice and minimise the diffusion length of Mg^2+^ ions in it.^11,12,14,18–20,23,25,26^ Recently, structured MgMn_2_O_4_ (theoretical discharge capacity: 270 mA h g^−1^) with continuous three-dimensional (3D) macro- and mesopores has been synthesised^20,23^ by a propylene-oxide-driven sol–gel method.^27–29^ Because of the small particle size (∼10 nm), high surface area (∼100–300 m^2^ g^−1^), and controlled bimodal pore size distribution in the micrometre (1–10 μm) and nanometre (10–100 nm) regions, cathodes of the material prepared by the sol–gel method outperform those of conventionally prepared MgMn_2_O_4_. Very recently, this structured MgMn_2_O_4_ has been used as the cathode active material in coin-type full cells operated at room temperature.^23^ While promising, the hydrophilicity of the structured MgMn_2_O_4_ limits its practical application in RMB fabrication: uniform slurry-coated cathodes are essential components, which cannot be prepared easily because of the tendency of the structured MgMn_2_O_4_ to aggregate in non-aqueous solvents like 1-methyl-2-pyrrolidone (NMP).

Side reactions during charging are common for transition metal oxide cathodes and present another challenge to their application in RMBs. Side reactions, such as the oxidative decomposition of organic electrolytes on the cathode surface, are usually associated with the high catalytic activities of transition metals^17^ and have been suppressed effectively by incorporating inert cations (*e.g.* Fe) into the spinel host lattice,^17^ or by coating cathode materials with less reactive oxides like V_2_O_5_.^21^

This study was aimed at developing a facile technique to functionalise the surface of the structured MgMn_2_O_4_ and suppress both water adsorption and undesirable electrochemical side reactions, while retaining the discharge capacity. Organic phosphate compounds were selected because of their ability to readily form strong chemical bonds with transition metal ions.^30–34^ The high selectivity of the functionalisation is expected to result in the formation of thin uniform monolayer that minimises the hindrance to the insertion and extraction of Mg^2+^ ions and passivates the active sites of side reactions. Phenylphosphonic acid was selected as a model compound containing hydrophobic functional groups. Additionally, anchoring phenyl groups to the surface of active materials is attractive, as π interactions between the phenyl groups and carbon-based electrically conductive supports, such as carbon nanotubes (CNTs), help strengthen the contact between them, potentially leading to an increase in the fraction of active material.

## Experimental procedure

The structured MgMn_2_O_4_ powder was prepared following a reported procedure.^20^ Stoichiometric amounts of magnesium and manganese chlorides (18 mmol in total) and citric acid (18 mmol, Fujifilm Wako Pure Chemical) were dissolved in 20 mL of ethanol, and propylene oxide (12 mL, Kanto Chemical) was added. The resulting metal–organic complex gel was maintained for 1 day at 25 °C, washed with ethanol and acetone to remove byproducts, and subjected to sequential solvent exchange with acetone and cyclohexane three times in 3 days. The resulting wet gel was freeze-dried using liquid nitrogen and heat treated for 5 h at 300 °C in air. The specific surface area of the powder evaluated by the Brunauer–Emmett–Teller (BET) method using a nitrogen adsorption isotherm was ∼100 m^2^ g^−1^. Ammonium phenylphosphonate (PhPO(ONH_4_)_2_) was obtained by adding excess aqueous ammonia (10 wt%) to an aqueous solution of phenylphosphonic acid (Tokyo Chemical Industry) and drying the solution at 80 °C. The resulting ammonium phenylphosphonate (1 mmol) was dissolved in 10 g of methanol, along with the structured MgMn_2_O_4_ powder (2.5 mmol), and the mixture was stirred for 3 h at room temperature to functionalise the MgMn_2_O_4_ with phenylphosphonate groups. The resulting suspension was centrifuged, washed twice with methanol, and dried at 60 °C in air to provide the phenylphosphonate-functionalised MgMn_2_O_4_ as a powder. The resulting samples were evaluated by powder X-ray diffraction (XRD, SmartLab, Rigaku) and Fourier-transform infrared (FT-IR) spectrometry (FT/IR-4600, JASCO) using an attenuated total reflection (ATR) unit with a diamond prism. Thermogravimetry and differential thermal analysis (TG-DTA, DTG-60, Shimadzu) were carried out at a heating rate of 5 K min^−1^ in air.

Dry composite cathodes were prepared by mixing the pristine or surface-functionalised MgMn_2_O_4_ powder, acetylene black (AB, Denka; electrically conductive support), and poly(tetrafluoroethylene) (PTFE, Du Pont-Mitsui Fluorochemicals; binder) at a weight ratio of 60 : 30 : 10, and the composite (∼2 mg) was pressed on a Pt mesh. Electrochemical measurements of the composite cathode were conducted at 100 °C in an Ar-filled glovebox with a three-electrode cell using a Mg ribbon (99.9%, Fujifilm Wako Pure Chemical) as the counter electrode, and Ag wire immersed in a triglyme (G3, Kanto Chemical) solution of 0.01 mol dm^−3^ AgNO_3_ (Kanto Chemical) and 0.1 mol dm^−3^ magnesium bis(trifluoromethanesulfonyl)amide (Mg[TFSA]_2_, Kishida Chemical) as the reference electrode. The solvated ionic liquid solution, 0.3 mol dm^−3^ [Mg(G4)][TFSA]_2_/[C_3_mPyr][TFSA],^35,36^ was prepared from tetraglyme (G4, Kishida Chemical), Mg[TFSA]_2_, and 1-methyl-1-propylpyrrolidinium bis(trifluoromethanesulfonyl)amide ([C_3_mPyr][TFSA], Tokyo Chemical Industry), and used as the electrolyte solution. Galvanostatic charge–discharge tests were carried out using an electrochemical analyser (HZ-Pro, Hokuto Denko) at 10 mA g^−1^ in the potential range from −1.6 to 0.6 V *vs.* Ag/Ag^+^ (from 1.0 to 3.2 V *vs.* Mg/Mg^2+^). It was initiated from the discharge step, and the charge capacity was restricted to 135 mA h g^−1^ (half of the theoretical capacity of MgMn_2_O_4_) to minimise the undesirable oxidative decomposition of the electrolyte solution.

Slurries to fabricate coated electrodes were prepared by mixing the MgMn_2_O_4_ powder, carbon nanotube (CNT, Cnano; electrically conductive support), and poly(vinylidene difluoride) (PVDF, Kureha; binder) at a weight ratio of 93 : 4 : 3 in NMP (Fujifilm Wako Pure Chemical). The slurry was applied on an Al foil and dried at 80 °C overnight under vacuum. The resulting slurry-coated cathode (*ϕ* 9.5 mm) was encapsulated in a 2032-type coin cell using a Mg–Al–Zn alloy plate (AZ31, Nippon Kinzoku, 3 wt% Al, 1 wt% Zn, *ϕ* 9.5 mm, 44 μm thick) as the anode, and glass filter paper (GA-55, Advantec) as the separator. A 0.3 mol dm^−3^ G3 solution of magnesium tetrakis(hexafluoroisopropyloxy)borate (Mg[B(hfip)_4_]_2_) was chosen as the electrolyte solution because of the low overpotentials for the Mg anode dissolution and deposition.^37–39^ Galvanostatic charge–discharge tests were performed using a battery testing system (HJ1020mSD8, Hokuto Denko) at 25 °C and 5 mA g^−1^ in the 0.1–4.0 V potential range, and the charge capacity was restricted to 135 mA h g^−1^. The slurry-coated cathodes were also characterised by scanning electron microscopy (SEM; JSM-6490A, JEOL) and energy-dispersive X-ray spectroscopy (EDS; JED-2300, JEOL).

## Results and discussion

**Fig. 1:**
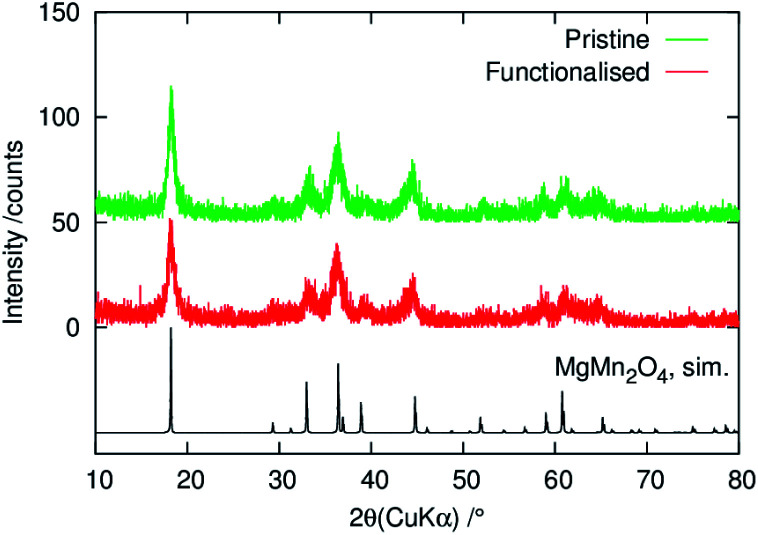
Powder XRD patterns of the pristine and surface-functionalised MgMn_2_O_4_ powders. Simulated pattern was calculated using RIETAN-FP^40^ and the structural parameters of MgMn_2_O_4_ reported in ref. 41.

Fig. 1 shows powder XRD patterns of pristine and phenylphosphonate-functionalised MgMn_2_O_4_. The observed patterns were essentially identical to the patterns for pristine samples reported previously.^20,23^ The broadening of diffraction peaks indicate nanocrystalline nature of the samples. The surface functionalisation did not change the diffraction pattern.

**Fig. 2:**
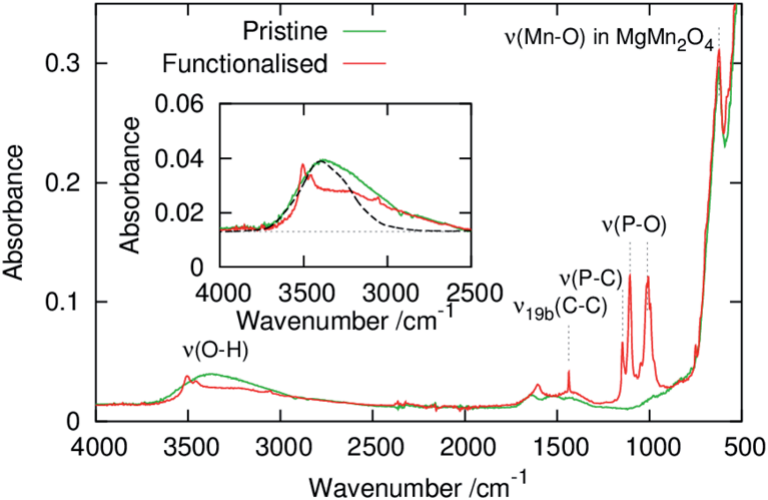
ATR-FT-IR spectra of the pristine and surface-functionalised MgMn_2_O_4_ powders. The inset shows magnified spectra and absorption spectrum of water (dashed line) taken from ref. 46.

Fig. 2 shows ATR-FT-IR spectra of powders of the pristine and phenylphosphonate-functionalised MgMn_2_O_4_. An absorption band originating from the Mn–O stretching mode of MgMn_2_O_4_ was observed at ∼650 cm^−1^.^42,43^ After surface functionalisation with phenylphosphonate groups, several new bands appeared. From the similarity of the spectra between this sample and related phenylphosphonate-functionalised transition metal oxides, absorption bands at ∼1010 and ∼1105 cm^−1^ were attributed to the P–O stretching modes.^30,31,34^ Sharp absorption bands at ∼1146 and ∼1438 cm^−1^ were assigned to the phenylphosphonate P–C stretching and *ν*_19b_ C–C ring modes, respectively.^34,44^ The absence of the P=O double bond stretching mode peak at 1200–1250 cm^−1^ suggests that the phenylphosphonate groups are covalently bonded to the MgMn_2_O_4_ surface and that the P=O bonds are converted to P–O–(Mn,Mg) bonds.^30,31,34^

A broad peak at 2500–3800 cm^−1^ was observed in the pristine MgMn_2_O_4_, which was ascribed to an O–H stretching mode with a shape comparable to that of water confined in mesopores.^45^ The component at wavenumbers below ∼3000 cm^−1^, which is absent in bulk liquid water,^46^ suggests the presence of structured water, *i.e.*, an ice-like hydrogen bonding network between water molecules and pore walls. These observations confirm significant water adsorption properties in mesopores of the pristine MgMn_2_O_4_. Surface functionalisation resulted in reduced intensity of the broad absorption centred at ∼3300 cm^−1^ and the appearance of a narrow absorption band at ∼3500 cm^−1^. The narrow band was assigned to isolated OH groups, which, when coupled with the shift to higher frequencies, indicates a weakening in hydrogen bonding^47^ caused by the partial desorption of physisorbed water molecules and isolation of surface OH groups.

**Fig. 3:**
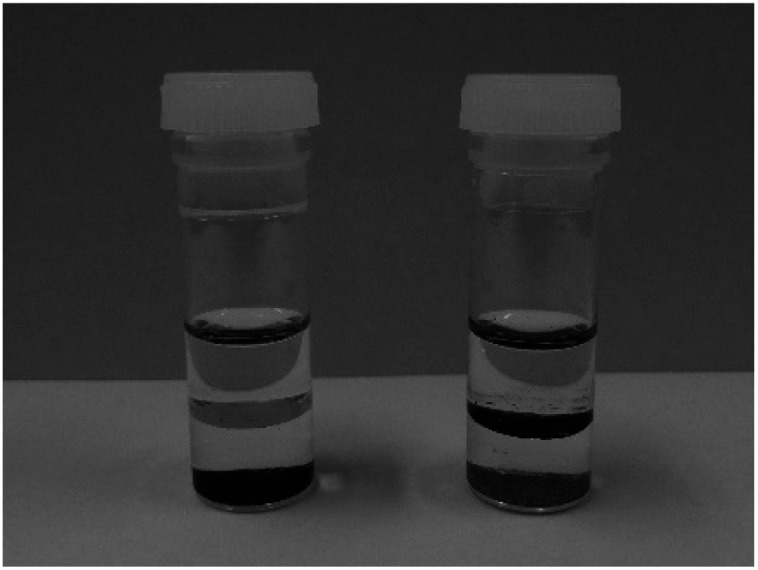
Pristine (left) and surface-functionalised (right) MgMn_2_O_4_ powders dispersed in a biphasic mixture of water (bottom layer) and toluene (top layer).

To visualise the effect of surface functionalisation, samples were dispersed in a water–toluene biphasic mixture as shown in Fig. 3. The pristine MgMn_2_O_4_ was precipitated at the bottom of the water layer. In contrast, the surface-functionalised MgMn_2_O_4_ was mostly suspended at the bottom of the toluene layer. These results confirmed that the surface of the structured MgMn_2_O_4_ was rendered hydrophobic after functionalisation with phenylphosphonate groups.

**Fig. 4:**
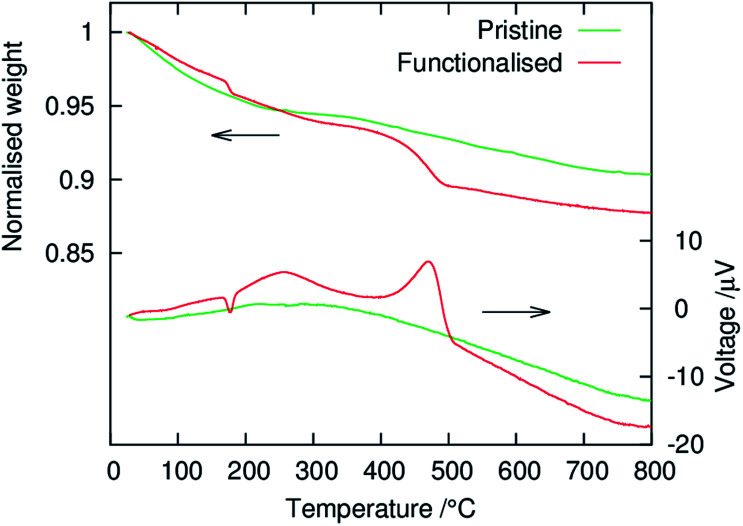
TG-DTA curves of the pristine and surface-functionalised MgMn_2_O_4_ powders.

The coverage of phenylphosphonate groups on the structured MgMn_2_O_4_ surface was evaluated using TG-DTA, and the results are shown in Fig. 4. In the pristine powder a two-step weight loss was observed, which can be explained by the desorption of physisorbed water (≲250 °C) and dehydration of OH groups along the MgMn_2_O_4_ grain growth (≳350 °C). In the functionalised powder, the weight loss by water desorption below ∼150 °C was smaller than in the pristine powder, consistent with the ATR-FT-IR results shown in Fig. 1. However, exothermic peaks associated with the combustion of organic substances were observed at higher temperatures. The weight loss at ∼450 °C (∼4%) was attributed to the thermal decomposition of phenyl groups. We assumed that this weight loss was associated with the conversion of C_6_H_5_PO_2_ to PO_5/2_, and the residue at 800 °C was formally represented as MgMn_2_O_4_·*x*PO_5/2_. The stoichiometry, *x*, of the phenylphosphonate groups with respect to MgMn_2_O_4_ was calculated to be *x* ≃ 0.14, which was equivalent to a surface density of ∼4 nm^−2^ for the phenylphosphonate groups when coupled with the MgMn_2_O_4_ surface area (∼100 m^2^ g^−1^). This equates to approximately half of the Mn surface density in MgMn_2_O_4_ (*e.g.* 6.1 nm^−2^ for the (001) face and 9.6 nm^−2^ for the (101) face), suggesting that there are approximately two Mn atoms per phenylphosphonate group on the surface. This is consistent with the bridged bidentate coordination mode known for phenylphosphonate- and phenylphosphinate-functionalised surfaces of atomically-flat transition metal oxides.^32–34^

**Fig. 5:**
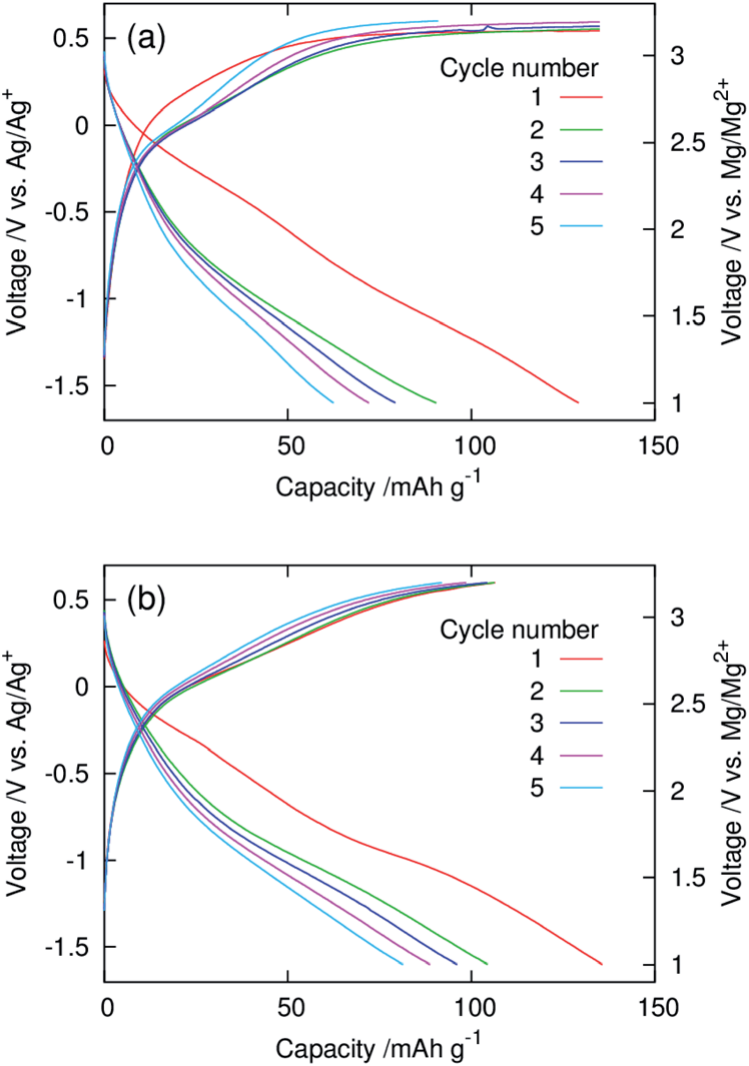
Galvanostatic charge–discharge curves of dry composite cathodes of the (a) pristine and (b) surface-functionalised MgMn_2_O_4_ prepared with the active material : AB : PTFE ratio of 60 : 30 : 10 (wt%) and recorded using a three-electrode setup at 100 °C.

Fig. 5 show galvanostatic charge–discharge curves of dry composite cathodes of the pristine and functionalised MgMn_2_O_4_ recorded at 100 °C. The current density was normalised with respect to the mass of the MgMn_2_O_4_ powder, which included the mass of phenylphosphonate groups in the functionalised one. The initial discharge capacities of the pristine and functionalised samples were ∼130 mA h g^−1^. After the second cycle, the functionalised sample exhibited higher discharge capacities, indicating that the functionalisation with phenylphosphonate groups increased the utility of MgMn_2_O_4_. In the pristine sample, charging in the first four cycles did not reach the cut-off (3.2 V *vs.* Mg/Mg^2+^) and ended at the predetermined capacity limit of 135 mA h g^−1^. In addition, the discharge capacity was significantly smaller than the corresponding charge capacity over each cycle, and poor coulombic efficiencies (∼0.46–0.67) were achieved. These observations indicate that significant side reactions occur during charging of the pristine sample. In contrast, in the functionalised sample, charging ended at the charge cut-off voltage, owing to the suppression of the side reactions, and the high coulombic efficiencies were obtained (∼0.83–0.98).

**Fig. 6:**
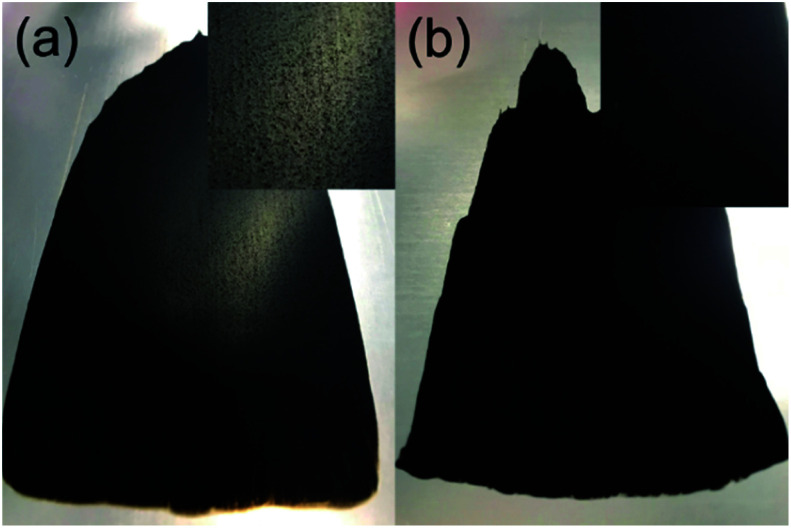
Photographs of composite slurry-coated cathodes of the (a) pristine and (b) surface-functionalised MgMn_2_O_4_ applied on Al foil, prepared with the active material : CNT : PVDF ratio of 93 : 4 : 3 (wt%). The widths of the coated area are ∼10 cm. The top-right inset in each figure shows ×2 magnified image of the coated area.

**Fig. 7:**
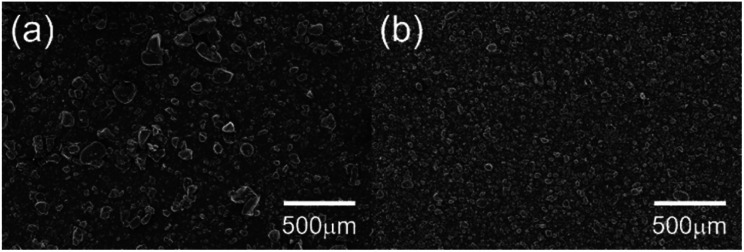
SEM images of the surfaces of the composite slurry-coated cathodes of the (a) pristine and (b) surface-functionalised MgMn_2_O_4_ applied on Al foil.

**Fig. 8:**
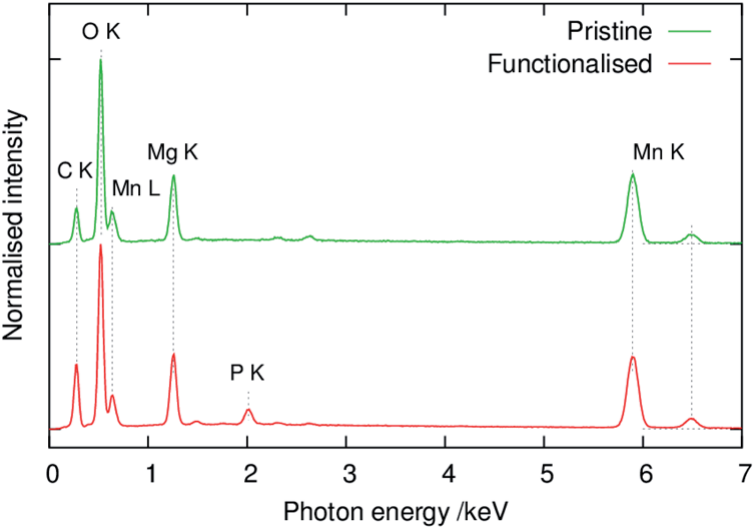
EDS spectra of composite slurry-coated cathodes shown in Fig. 7.

Fig. 6 shows images of the composite slurry-coated cathodes of the pristine and functionalised MgMn_2_O_4_ applied on Al foil. The cathode of the pristine sample was not smooth and exhibited MgMn_2_O_4_ aggregates, which were easily detached from the Al foil after drying. In contrast, the cathode of the functionalised sample was uniform, and its adhesion to the Al foil was good. Fig. 7 shows SEM images of the coated cathodes. The smoothness of the coated cathode of the functionalised MgMn_2_O_4_ was much better than that of the pristine MgMn_2_O_4_. Large particles seen in Fig. 7(a) were the aggregates of the pristine MgMn_2_O_4_ formed during slurry preparation because such large particles were scarce before mixing. Thus, the surface functionalisation improved the homogeneity of slurries, and it would be responsible for the smoothness and good adhesion of the coated cathodes of the functionalised MgMn_2_O_4_. Fig. 8 shows EDS spectra of the coated cathodes. The P K peak (2.01 keV) was seen in the functionalised MgMn_2_O_4_, confirming the presence of phenylphosphonates. In this cathode, CNTs were used as the electrically conductive support, and the fraction of active material was as high as 93 wt%, which is, to the best of our knowledge, the highest reported thus far in the field of RMBs.

**Fig. 9:**
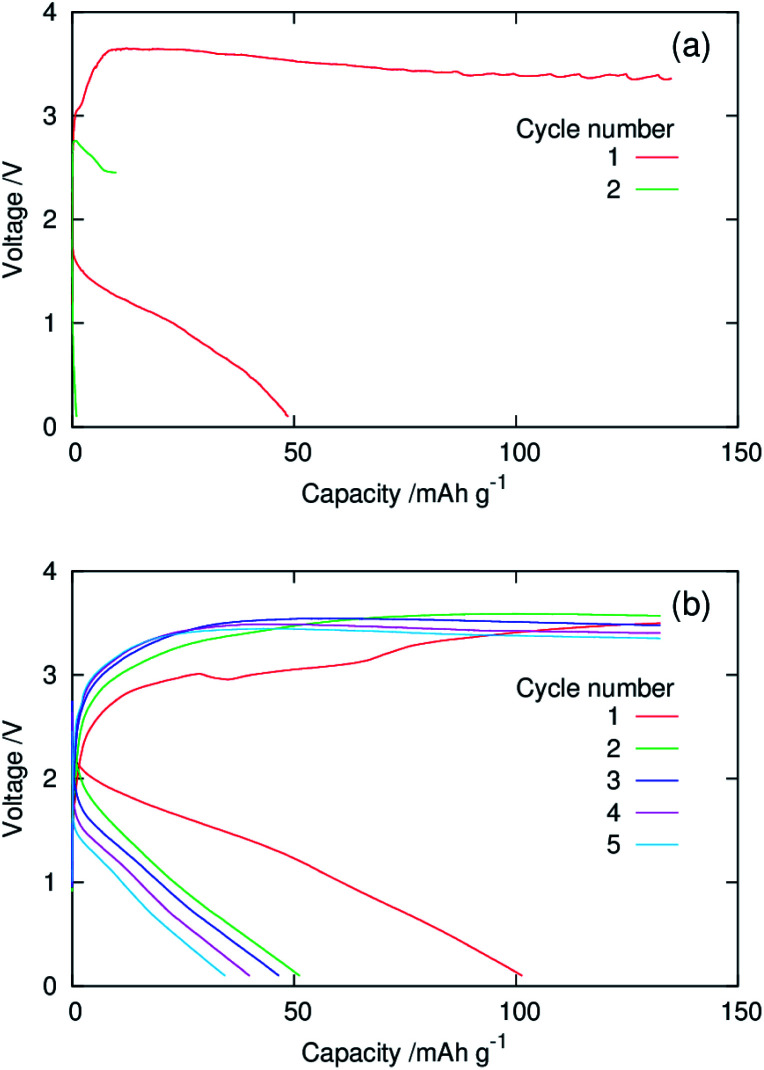
Galvanostatic charge–discharge curves of coin-type cells with the composite slurry-coated cathodes of the (a) pristine and (b) surface-functionalised MgMn_2_O_4_ shown in Fig. 6 and Mg–Al–Zn alloy anode recorded at 25 °C.

Fig. 9 shows the galvanostatic charge–discharge curves of the coin-type full cells with a slurry-coated cathode of the pristine or functionalised MgMn_2_O_4_ and a Mg–Al–Zn alloy anode recorded at 25 °C. The full cell with the cathode of the pristine sample (∼3 μm thick, active material loading ∼0.70 mg cm^−2^) delivered an initial discharge capacity of ∼50 mA h g^−1^. However, the discharge capacity dropped abruptly to ∼1 mA h g^−1^ in the second cycle, and the cell broke during subsequent charging. We suggest that the degradation was the result of side reactions during charging, which may have been considerable during the gradual voltage decay at ≳10 mA h g^−1^ in the initial charge step. The poor cyclability and large polarisation indicate insufficient current collection arising from inhomogeneous mixing between the MgMn_2_O_4_ and CNTs. The weak adhesion between the cathode and the Al foil may also contribute to the degradation of cyclability.

In contrast, the full cell of the surface-functionalised MgMn_2_O_4_ (∼5 μm thick, active material loading ∼0.74 mg cm^−2^) delivered an initial discharge capacity of ∼100 mA h g^−1^, even at a low CNT fraction (4 wt%). The initial discharge capacity agreed well with the value (∼100 mA h g^−1^) expected for the pristine structured MgMn_2_O_4_ with the BET surface area of ∼100 m^2^ g^−1^, when evaluated at 25 °C using coin-type full cells with dry composite cathodes at the active material : AB : PTFE weight ratio of 60 : 30 : 10.^23^ These results demonstrate that the phenylphosphate functionalisation of the structured MgMn_2_O_4_ surface significantly improves the current collection efficiency in slurry-coated cathodes with high active material fractions. The improvement in the electrical contact between the surface-functionalised MgMn_2_O_4_ and CNTs is attributed to π interactions between phenyl groups and carbon surfaces, increasing their affinity. Surface functionalisation also reduced polarisation during cycling. The suppression of side reactions by surface functionalisation would be another major contributing factor to such improvements seen in the electrochemical properties. The discharge capacity decreased to ∼50 mA h g^−1^ after the second cycle. This capacity fading behaviour is also in accordance with that of the dry composite cathodes of the pristine structured MgMn_2_O_4_ in coin-type full cells.^23^ The charge curves of the coin-type cell exhibited plateau originating from the oxidative decomposition of electrolytes. In this experiment, a high cut-off voltage (4 V) was necessary to complete charging by compensating the overpotential of Mg deposition on the Mg–Al–Zn alloy anode, which fluctuated during cycling.^23^ Hence, it was difficult to maintain the cathode potential of the coin-type cell below 3.2 V *vs.* Mg/Mg^2+^, at which the electrolyte decomposition on the functionalised MgMn_2_O_4_ was slow (Fig. 5(b)). This problem may be overcome by the improvement of anodes and electrolytes to decrease the overpotential of Mg deposition.

Thus, the phenylphosphonate functionalisation technique investigated is a highly effective strategy to ensure the fabrication of uniform slurry-coated cathodes of nanostructured hydrophilic transition-metal-based electrode active materials of high surface areas at high active material fractions, by preventing common aggregation issues in non-aqueous slurries for such materials.

## Conclusions

A facile method for the surface functionalisation of transition metal oxide cathodes with phenylphosphonates has been developed, making use of the strong affinity between transition metal ions and phosphonate groups. The cathode active mateials were rendered hydrophobic by the presence of phenyl groups at the surface, and their use in RMBs was investigated. This technique suppressed the water adsorption of the hierarchically structured MgMn_2_O_4_, a promising high-voltage cathode active material for RMBs because of its high surface area (≳100 m^2^ g^−1^) and small particle size (∼10 nm) that facilitate the insertion and extraction of Mg^2+^ ions. This treatment reduced the aggregation of the structured MgMn_2_O_4_ in NMP-based non-aqueous composite cathode slurries employing CNT as an electrically conductive support, and enabled the production of uniform slurry-coated cathodes with improved contact between MgMn_2_O_4_ and CNTs. In addition, the surface functionalisation suppressed side reactions during charging and significantly increased the coulombic efficiency, while maintaining the discharge capacity. A coin-type full cell consisting of a slurry-coated cathode with active material fractions up to 93 wt%, a Mg–Al–Zn alloy anode, and a Mg[B(hfip)_4_]_2_ electrolyte delivered an initial discharge capacity of ∼100 mA h g^−1^ at 25 °C. This surface functionalisation technique is attractive for the development of practical slurry-coated cathodes of nanosized hydrophilic transition metal oxides for RMBs.

## Conflicts of interest

There are no conflicts to declare.

## Supplementary Material
